# Monitoring of the invasion of *Spartina alterniflora* from 1985 to 2015 in Zhejiang Province, China

**DOI:** 10.1186/s12898-020-00277-8

**Published:** 2020-02-06

**Authors:** Nan Li, Longwei Li, Yinlong Zhang, Ming Wu

**Affiliations:** 1 Collaborative Innovation Center of Sustainable Forestry in Southern China of Jiangsu Province, Nanjing Forestry University, Nanjing, 311300 China; 2 School of Environmental & Resource Sciences, Zhejiang Agriculture and Forestry University, Hangzhou, 311300 China; 3 Institute of Subtropical Forestry Research, Hangzhou, 311400 China

**Keywords:** Dynamic change, Expert knowledge, Invasive plants, Landsat images, *Spartina alterniflora*

## Abstract

**Background:**

*Spartina alterniflora* is an invasive plant on the coast of China that replaces native vegetation and has a serious negative impact on local ecosystems. Monitoring the spatial distribution of *S. alterniflora* and its changes over time can reveal its expansion mechanism, which is crucial for the management of coastal ecosystems. The purpose of this study was to map the distribution of *S. alterniflora* in Zhejiang Province from 1985 to 2015 using a time series of Landsat TM/OLI images and analyze the temporal and spatial patterns of expansion of this species.

**Results:**

After analyzing the distribution of coastal vegetation, the vegetation index was calculated based on Landsat images for 4 years (1985, 1995, 2005 and 2015). According to a threshold determined based on expert knowledge, the distribution of *S. alterniflora* in Zhejiang Province was extracted, and the temporal and spatial changes in the distribution of *S. alterniflora* were analyzed. The classification accuracy was 90.3%. *S. alterniflora* has expanded rapidly in recent decades after being introduced into southern Zhejiang. Between 1985 and 2015, *S. alterniflora* increased its area of distribution by 10,000 hm^2^, and it replaced native vegetation to become the most abundant halophyte in tidal flats. Overall, *S. alterniflora* expanded from south to north over the decades of the study, and the fastest expansion rate was 463.64 hm^2^/year, which occurred between 1995 and 2005. *S. alterniflora* was widely distributed in the tidal flats of bays and estuaries and expanded outward as sediment accumulated.

**Conclusions:**

This study reveals the changes over time in *S. alterniflora* cover in Zhejiang and can contribute to the control and management of this invasive plant.

## Background

*Spartina alterniflora* Loisel. is a perennial halophyte that is native to the Atlantic and Gulf coasts of North America and predominates in local salt marshes [1]. *S. alterniflora* is generally considered beneficial in ecological restoration because of its well-developed underground structure, high salt tolerance, high reproductive capacity and rapid growth [2]. For this reason, in December 1979, *S. alterniflora* was intentionally introduced as an ecological engineering species into China for sediment accumulation, land reclamation and saline soil amelioration [3].

Experiments have shown that *S. alterniflora* can perform ecological functions and provide economic benefits [2, 4–6]. Coastal stabilization and land reclamation are the most important ecological functions of this species. A trial planting of *S. alterniflora* was employed to solve river-shore crumbling near a sluice in Zhejiang Province in 1986. The planting successfully solved this problem, costing only 800 Yuan, and withstood typhoons and floods [2]. The *S. alterniflora* marsh on the canal bank prevented more than 100,000 m^3^ of sediment per year from migrating downstream [3]. In addition, Shen et al. [7] proved the efficacy of this plant for saline soil amelioration. Furthermore, *S. alterniflora* is used as animal fodder and fish feed [2, 8, 9] and can be used to produce green manure and biomineral liquids [1].

Since its introduction, *S. alterniflora* has greatly expanded in distribution in the coastal salt marshes of China [1, 10]. An increasing number of studies have demonstrated that although this exotic species can be beneficial, it has negative impacts on native coastal ecosystems [11–14]. Once established, *S. alterniflora* quickly expands on bare beaches and competes with native plants, invading their habitats and replacing them. Areas of *Scirpus mariqueter* Wang et Tang and *Phragmites australis* Trin. ex Steud. and young mangrove swamps have been invaded by *S. alterniflora*. Research shows that the species richness of benthic macroinvertebrates in *S. alterniflora* swamps is reduced because its invasion alters the physicochemical properties of the sediment [15]. Some endangered birds are threatened because the density of *S. alterniflora* communities is too high and the altered habitats are no longer suitable for them [16]. Therefore, the habitats of native plants, birds and benthic animals are affected by *S. alterniflora*, and biodiversity is reduced [17, 18]. In addition, the invasion of *S. alterniflora* impedes the development of local aquaculture and tourism and hinders water-based transportation [19, 20]. The significant negative impacts of this species have far exceeded its ecological functions and economic benefits [12, 21, 22]. *S. alterniflora* was listed as one of the top 16 invasive alien species by the State Environmental Protection Administration of China in 2003 [1, 23].

Biological invasion has a strong influence on Earth’s ecosystems and is considered one of the three most pressing environmental issues [24–26]. *S. alterniflora* threatens local ecosystems and causes extremely large losses to the regional economy [11]. At present, from Guangxi Province (21° 33′ N, 108° 08′ E) to Liaoning Province (40° 20′ N, 122° 35′ E), *S. alterniflora* can be found in most of China’s coastal areas [10, 27]. According to the Marine Environment Quality Bulletin of China, this species invaded 12,400 ha of China’s coastal areas in 2006. Jianbo Lu [1] stated that it covered a total area of 34,178 ha in 2012. Hence, the invasion of *S. alterniflora* has become a hot topic for ecologists and biologists at home and abroad.

Over the years, scholars have conducted many studies on the invasion of *S. alterniflora* in China’s coastal areas. However, there are few studies on the monitoring of changes in *S. alterniflora* distribution, with most such studies limited to monitoring the distribution of *S. alterniflora* in a given year. The lack of long-term sequential monitoring of *S. alterniflora* not only limits our understanding of the invasion mechanism but also restricts the decision-making of government departments. To better understand the expansion mechanism of *S. alterniflora* and prevent its further invasion, it is necessary to map its distribution and monitor its dynamic changes. Therefore, this study employed a time series of Landsat Thematic Mapper (TM)/Operational Land Imager (OLI) images to monitor the dynamic changes in *S. alterniflora* in the coastal areas of Zhejiang Province, China. Specifically, the spatial distribution of *S. alterniflora* in Zhejiang Province from 1985 to 2015 was mapped, the spatial and temporal heterogeneities of *S. alterniflora* expansion were analyzed, and the changes in *S. alterniflora* in bays and estuaries were analyzed. This study documents the temporal and spatial changes in *S. alterniflora* invasion on the eastern coast of China, providing important data for the ecological study of *S. alterniflora*.

## Methods

### Study area

**Fig. 1:**
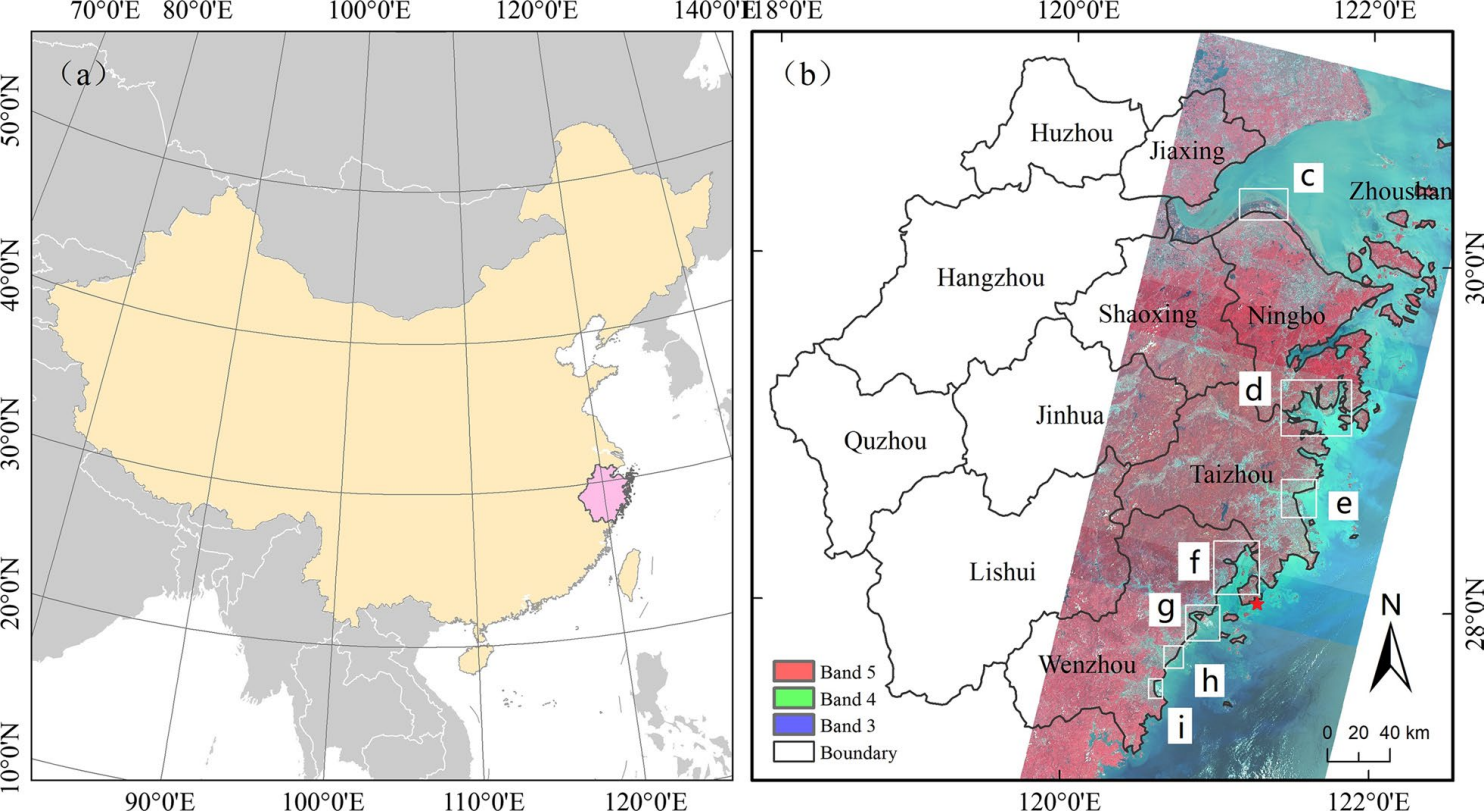
The location of the study area: **a** Zhejiang Province in eastern China; **b** the study area in the coastal area of Zhejiang Province and a false color composite of Landsat OLI imagery; **c**–**i** denote (in order) Hangzhou Bay, Sanmen Bay, Jiang River Estuary, Yueqing Bay, Ou River Estuary, Feiyun River Estuary and Ao River Estuary. The red star marks the first location where *S. alterniflora* was introduced. The administrative boundary was obtained from the Resource and Environment Data Cloud Platform of the Chinese Academy of Sciences (http://www.resdc.cn), and the map was designed by Nan Li

Zhejiang Province is located in eastern China (Fig. 1a). It has a subtropical monsoon climate with moderate annual temperatures and abundant sunshine and rainfall. Zhejiang has abundant marine resources, with a coastline of 6486.24 km, accounting for 20.3% of China’s coastline. According to the second wetland resource survey, the tidal flats are the largest natural wetland in the province, covering 1548.86 km^2^. The tidal flats are mainly distributed in Hangzhou Bay, Sanmen Bay and Yueqing Bay. In 1983, *S. alterniflora* was first planted in the tidal flats of Yuhuan County and then introduced to other places along the coast. Over the past 30 years, *S. alterniflora* has rapidly expanded and become the main salt marsh vegetation along the coast of Zhejiang Province.

### Data collection and preprocessing

The data collected in this study included remote sensing images, vector data such as administrative boundaries, field survey data and literature data. Remote sensing is an effective tool for monitoring changes in Earth’s surface and is suitable for coastal wetland monitoring and vegetation monitoring [27–30]. Since 1972, NASA has launched a number of Landsat series satellites, which are the most commonly used remote sensing data sources. Twenty-six scenes of Landsat TM/OLI images were obtained from the U.S. Geological Survey Global Visualization data server (https://glovis.usgs.gov/app). The principles of remote sensing image acquisition included (1) selecting images taken between May and November, which is the growth season of *S. alterniflora* and (2) selecting high-quality images without cloud cover. Table 1 summarizes the acquisition dates and sensor types of the remote sensing images used in this study.

**Table 1:** Dates and sensors for the remote sensing imagery used in this study

Year	Path/row			Sensors
	118/39	118/40	118/41	
1985	1985.07.15	1984.05.09	1984.04.23	TM
		1985.06.29	1986.05.31	
1995	1995.08.12	1995.03.05	1995.08.12	TM
	1995.11.16	1995.11.16	1995.11.16	
2005	2005.06.04	2005.06.04	2005.06.04	TM
	2005.11.27	2005.11.27	2005.11.27	
2015	2014.11.04	2014.11.04	2014.11.04	OLI
	2015.08.03	2015.08.03	2015.08.03	
	2016.07.20	2016.07.20	2016.07.20	

From May to November in 2015, field investigations were conducted in coastal areas of Zhejiang Province. The *S. alterniflora* community as determined visually in the field was marked on a Google Earth map with polygons. In addition, the positions of *S. alterniflora* plants were recorded using GIStar 710, a hand-held geographic positioning system device (South Surveying & Mapping Instrument Co., Guangzhou, China). Field surveys provide important location information for visual interpretation and classification of remote sensing images. A large number of Chinese and English research papers and statistical yearbooks were reviewed to obtain information about the distribution of *S. alterniflora*. In addition, detailed consultations were conducted with local people through field investigations.

All remote sensing images were preprocessed with ENVI 5.3 software, including radiometric calibration, atmospheric correction and image-to-image registration. The Fast Line-of-Site Atmospheric Analysis of Spectral Hypercubes [31] method was used for atmospheric correction. The latest image was used as the reference image to register images from other periods. More than 30 control points were selected for each registration, and the root mean square error was less than 0.5 pixels. All images were under Universal Transverse Mercator 50 (UTM-50) projection systems.

### Extracting the distribution of *S. alterniflora*

**Fig. 2:**
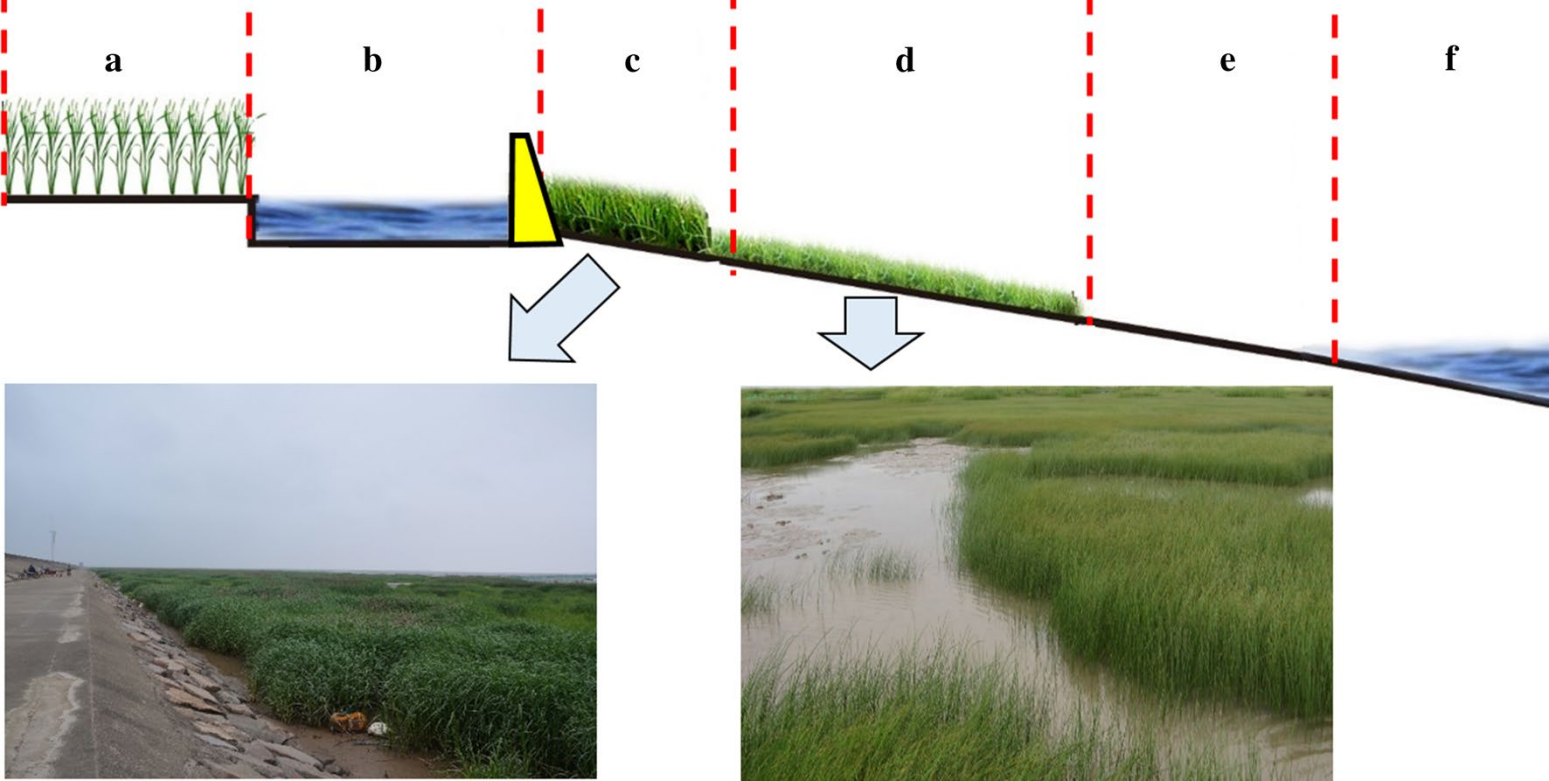
Different land types and main vegetation along the coast. **a**–**f** denote (in order) aquaculture ponds, farmland, *Spartina alterniflora, Scirpus mariqueter*, mud beach and sea water. The yellow trapezoid is a dike

Based on literature surveys and the field investigations, it was determined that *S. alterniflora* grows only in intertidal zones with high salinity and does not exist inland. In addition, there are dikes along the coast of Zhejiang Province that can prevent waves, which form an obvious artificial boundary between tidal flats and inland areas. *S. alterniflora* (Fig. 2c), *S.* *mariqueter* (Fig. 2d), mud beach (Fig. 2e) and sea water (Fig. 2f) are located outside the dikes, and aquaculture ponds (Fig. 2a) and farmland (Fig. 2b) are located inside the dikes. The coastline was used as a baseline to obtain a 2 km buffer area outside the dikes. In this area, the main plants are only *S. alterniflora* and *S. mariqueter*, facilitating the identification of *S. alterniflora* [27].

The growth environments and spectral characteristics of *S. alterniflora* and *S.* *mariqueter* are quite different. *S. alterniflora* grows vigorously and has high coverage, showing typical vegetation spectral characteristics. The plants of *S.* *mariqueter* are low, sparse, and often submerged by tides, thus exhibiting mixed spectral characteristics of vegetation, water and soil. The normalized difference vegetation index (NDVI), which can express vegetation characteristics, was calculated. Analysis revealed that during the growing season, the NDVI of *S. alterniflora* was higher than that of *S. mariqueter*, which was quite low. Therefore, according to their NDVI differences and previous studies [27, 29], an appropriate threshold was selected, and decision tree classification was used to extract *S. alterniflora*. Then, Google Earth images were used for postprocessing, unambiguously misclassified land types were modified, and the final spatial distribution of *S. alterniflora* was obtained. Images from 2010 and other periods were processed in the same way. According to the field survey, 300 verification points were randomly selected for verification, a confusion matrix was used to evaluate the performance of the classification, and overall accuracy was used to assess the classification accuracy.

### Detecting the change in *S. alterniflora* distribution

After extracting *S. alterniflora* from all images by ENVI 5.3 software, a distribution thematic map was constructed using ArcGIS 10.2 software, and the distribution changes were analyzed using the overlay function. The area of *S. alterniflora* within the administrative area of each city in each period was determined using ArcGIS 10.2 software. To better understand the expansion of *S. alterniflora*, based on the classification results of the previous step, the change in *S. alterniflora* was detected. In this study, the percentage (%) of the area and total area of *S. alterniflora* in different regions were calculated for various years. The total expansion area per decade was calculated, and the annual expansion area was defined as (X_j_ − X_i_)/(j − i), where X_i_ and X_j_ are the areas of *S. alterniflora* in the prior year i and the subsequent year j, respectively.

The present study focused on bay and estuary areas to explore the expansion patterns of *S. alterniflora* in different areas along the coast of Zhejiang Province. Bays are typically surrounded by land on three sides, exhibiting a U-shape or arc shape. The bays in Zhejiang mainly include Hangzhou Bay, Yueqing Bay and Sanmen Bay (Fig. 1). An estuary is the area where the river enters the sea, typically exhibiting an open, fan-shaped area and significant tidal phenomenon. The main estuaries in Zhejiang Province are Jiang River Estuary, Ou River Estuary, Feiyun River Estuary and Ao River Estuary (Fig. 1).

## Results

### Spatial distribution of *S. alterniflora* in Zhejiang Province

**Fig. 3:**
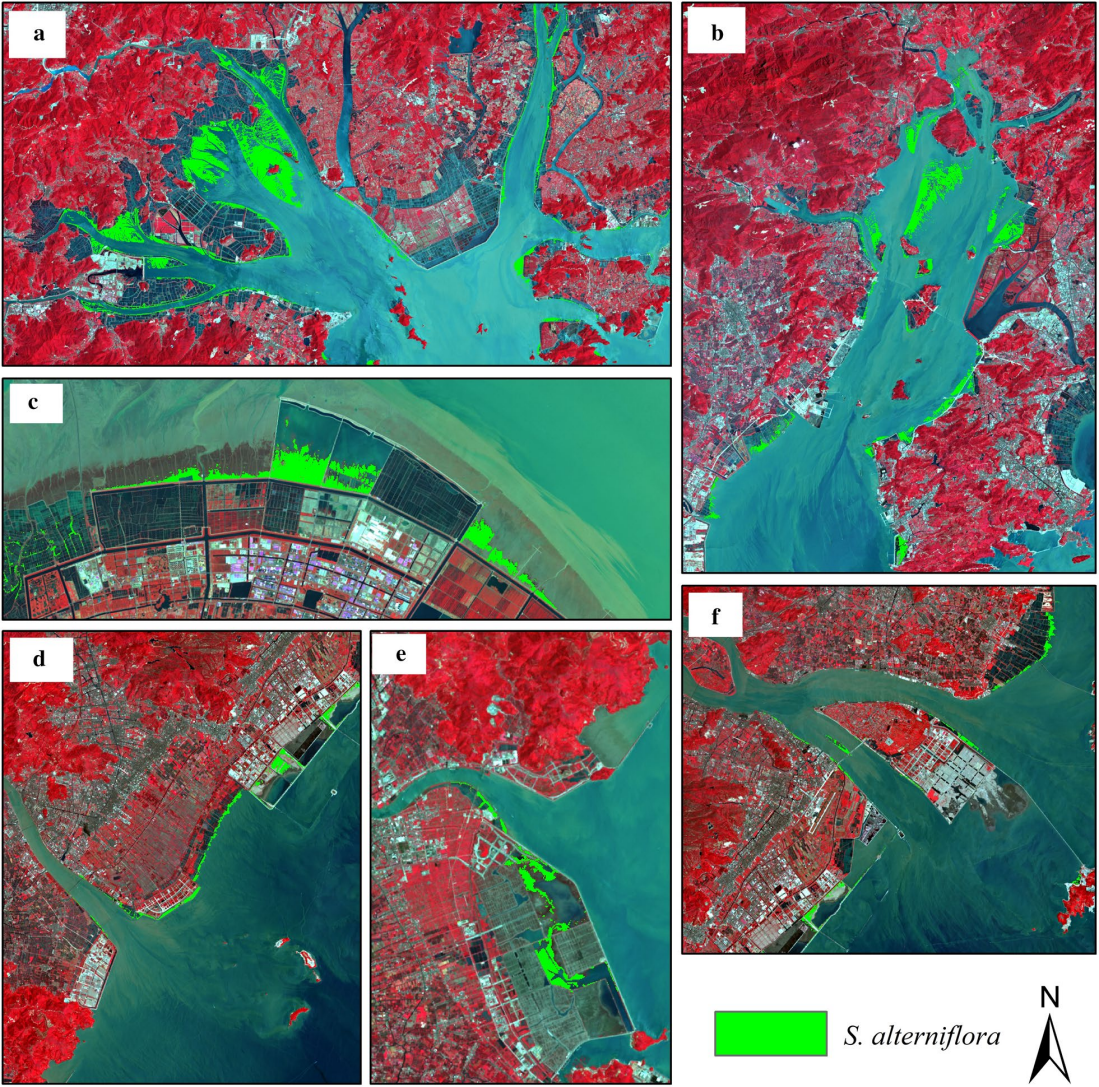
The spatial distribution of *S. alterniflora* in Zhejiang Province in 2015. **a** Sanmen Bay in Taizhou; **b** Yueqing Bay in Wenzhou; **c** Hangzhou Bay in Ningbo; **d** Feiyun River Estuary in Wenzhou; **e** Ao River Estuary in Wenzhou; **f** Ou River Estuary in Wenzhou

According to the field survey data, the classification results were verified, and the confusion matrix was calculated. The results showed that the overall accuracy was 90.3%, and the kappa coefficient was 0.91, meeting the research requirements. In 2015, *S. alterniflora* was estimated to cover more than 10,000 hm^2^, from the northernmost point (Jiaxing) to the southernmost point (Wenzhou) along the coastline (Fig. 3). The species was mainly concentrated in the bays, estuaries, and ports, such as Hangzhou Bay, Sanmen Bay, Yueqing Bay, Feiyun River Estuary, Ao River Estuary, and Xiangshan Port. There were fewer *S. alterniflora* plants on the islands.

Table 2 summarizes the area of *S. alterniflora* in the coastal areas of Zhejiang from 1985 to 2015. *S. alterniflora* was first introduced and planted on the southern coast of Zhejiang Province in 1983; it did not extensively expand and could not be identified from the Landsat imagery until 1985. This delay occurred because of the coarse spatial resolution (30*30 m) of the Landsat TM imagery and because vegetation with an area of less than 900 m^2^ could not be accurately identified from the images.

**Table 2:** *Spartina alterniflora* in Zhejiang Province from 1985 to 2015

	1985		1995		2005		2015	
	hm^2^	%	hm^2^	%	hm^2^	%	hm^2^	%
Zhoushan	–	–	–	–	1.71	0.03	83.07	0.83
Jiaxing	–	–	96.57	5.28	263.97	4.08	322.11	3.21
Ningbo	–	–	569.52	31.16	3706.47	57.34	5425.83	54.05
Taizhou	–	–	64.80	3.55	916.65	14.18	2760.03	27.50
Wenzhou	–	–	1096.74	60.01	1575.27	24.37	1447.11	14.42
Zhejiang Province	–	–	1827.63	100.00	6464.07	100.00	10,038.15	100.00

By 1995, the area of *S. alterniflora* had expanded to 1827.63 hm^2^, mainly concentrated in Wenzhou, with an area of 1096.74 hm^2^, accounting for 60.01% of the total area. In 2005, the total area of *S. alterniflora* was 6464.07 hm^2^, and the species was mainly distributed in Ningbo, with an area of 3706.47 hm^2^. By 2015, the area of *S. alterniflora* reached 10,038.15 hm^2^, and the species was mainly distributed in Ningbo and Taizhou.

### Spatial and temporal variability of *S. alterniflora* expansion

*S. alterniflora* spread widely along the coast from 1985 to 2015, with the area increasing by 10,000 hm^2^ and an average annual growth area of 334.61 hm^2^ (Table 3). From 1985 to 1995, the expansion of *S. alterniflora* was relatively slow, with an annual expansion area of 182.76 hm^2^. Then, it expanded extensively during the decade from 1995 to 2005, with an average annual growth area of 463.64 hm^2^. After 2005, the growth rate decreased to 357.41 hm^2^. *S. alterniflora* significantly damaged the composition of local ecosystems and the landscape structure. The local government used several methods to control its growth. After 2005, *S. alterniflora* was partially under control, and the annual expansion area was reduced to 357.41 hm^2^.

**Table 3:** Analysis of the expansion of *S. alterniflora* from 1985 to 2015

District	Expanded area per 10 years (hm^2^)			1985–2015
	1985–1995	1995–2005	2005–2015	
Zhoushan	0.00	1.71	81.36	83.07
Jiaxing	96.57	167.40	58.14	322.11
Ningbo	569.52	3136.95	1719.36	5425.83
Taizhou	64.80	851.85	1843.38	2760.03
Wenzhou	1096.74	478.53	− 128.16	1447.11
Zhejiang Province	1827.63	4636.44	3574.08	10,038.15

District	Annual expansion area (hm^2^/year)			1985–2015
	1985–1995	1995–2005	2005–2015	
Zhoushan	0.00	0.17	8.14	2.77
Jiaxing	9.66	16.74	5.81	10.74
Ningbo	56.95	313.70	171.94	180.86
Taizhou	6.48	85.19	184.34	92.00
Wenzhou	109.67	47.85	− 12.82	48.24
Zhejiang Province	182.76	463.64	357.41	334.61

The expansion of *S. alterniflora* exhibited spatial and temporal variability in different coastal areas. In the past 30 years, the area of *S. alterniflora* in Ningbo has increased the most, expanding by 5425.83 hm^2^. In the middle decade, *S. alterniflora* had the fastest expansion rate of 313.7 hm^2^ per year. *S. alterniflora* in Wenzhou grew fastest in the first decade and then expanded at a slower rate and experienced negative growth in the last decade. The expansion of *S. alterniflora* in Taizhou was severe, and the annual expansion area increased each year. The expansion trend of *S. alterniflora* in Jiaxing was similar to that in Wenzhou, and its expansion rate declined in the last decade. *S. alterniflora* had the smallest area in Zhoushan, but it had been increasing over the last 30 years.

### The expansion pattern of *S. alterniflora* in typical areas

**Fig. 4:**
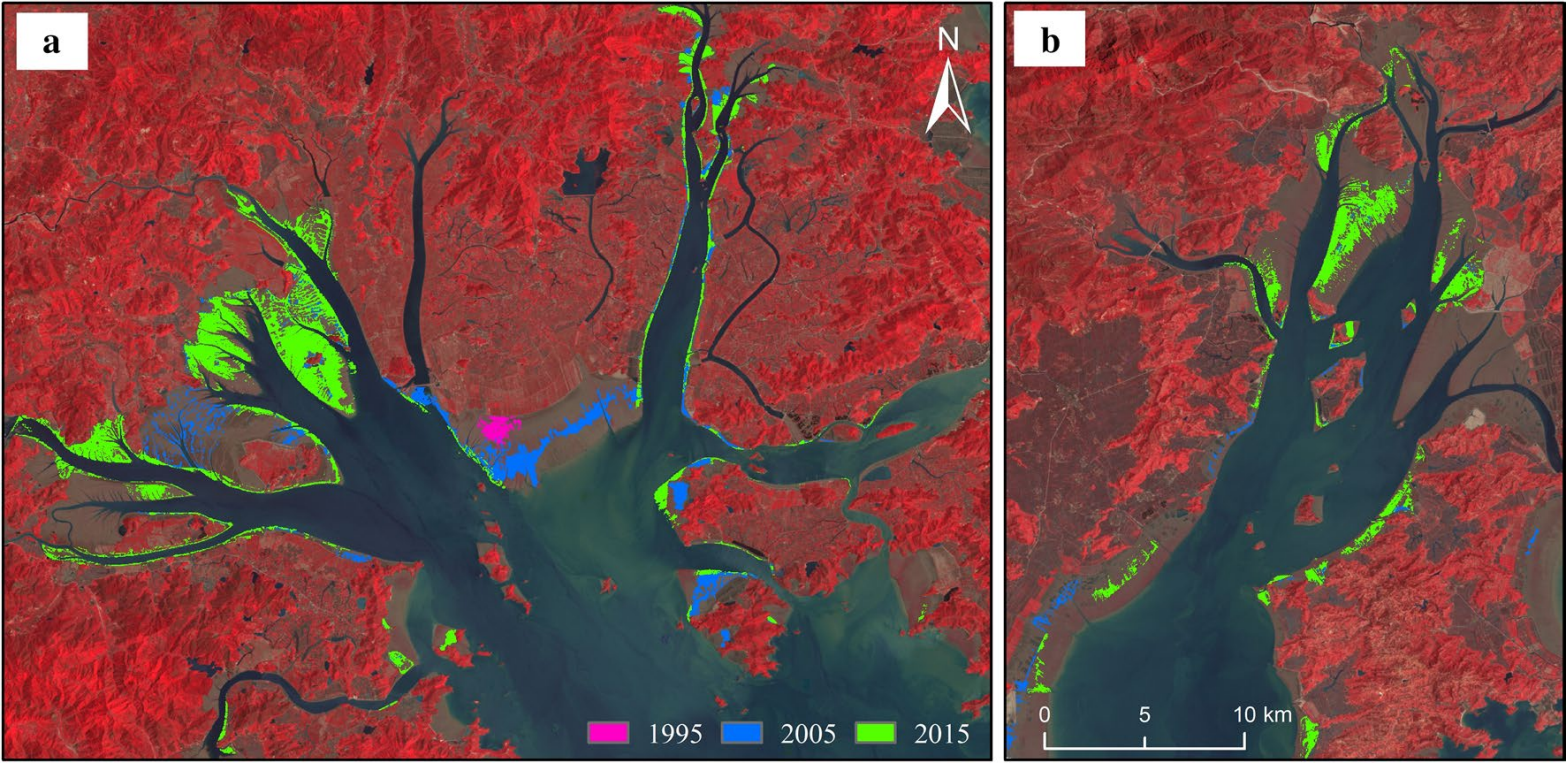
The expansion of *S. alterniflora* in Sanmen Bay (**a**) and Yueqing Bay (**b**). Red, blue and green represent the distribution of *S. alterniflora* in 1995, 2005 and 2015, respectively

*S. alterniflora* had different expansion patterns in the bays and estuaries. Yueqing Bay (Fig. 4a) and Sanmen Bay (Fig. 4b) are typical semiclosed bays in the study area, which extend deep into the interior. The tidal range in the bay is wide, and the winds and waves in the bay are mild, which creates conditions suitable for the growth of *S. alterniflora*. In these areas, *S. alterniflora* was widely distributed in the tidal flats, and a small number of plants were distributed along the coastline.

**Fig. 5:**
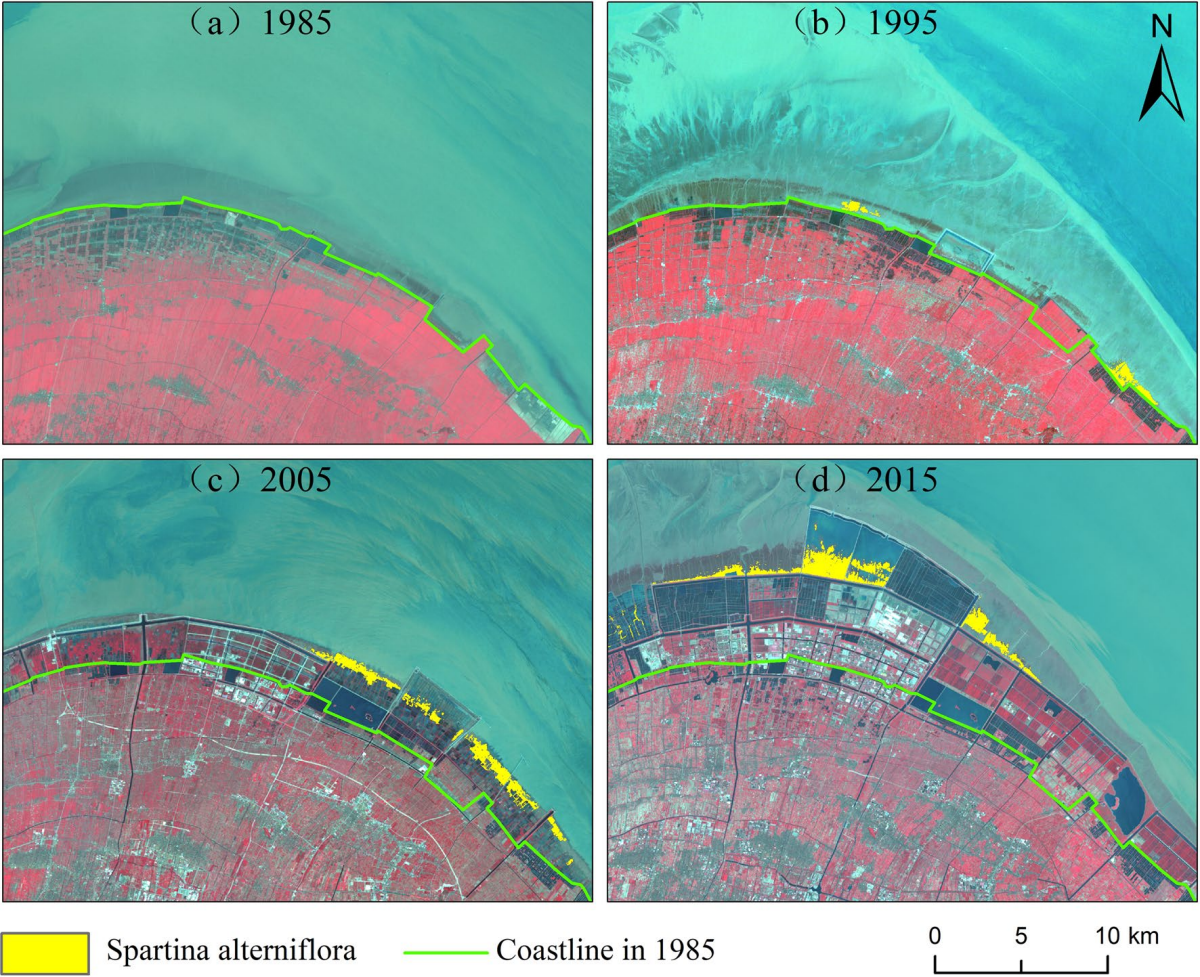
The expansion of *S. alterniflora* in Hangzhou Bay in recent decades

Hangzhou Bay is a trumpet-shaped bay located at the mouth of the Qiantang River. The suspended sediment content in the area is quite high. Due to the special terrain of the bay, the ocean current runs northward, causing erosion on the north bank and siltation on the south bank. Studies have shown that the south bank of Hangzhou Bay has expanded by 9 km in the past three decades. As shown in Fig. 5, as the coastline expanded each year, *S. alterniflora* expanded correspondingly, and its area increased. In 1985, there was no *S. alterniflora* in the area (Fig. 5a); in 1995, a small number of *S. alterniflora* plants were located on the southeastern shore (Fig. 5b). In 2005, *S. alterniflora* was distributed in strips (Fig. 5c). In 2015, *S. alterniflora* was widely distributed (Fig. 5d).

**Fig. 6:**
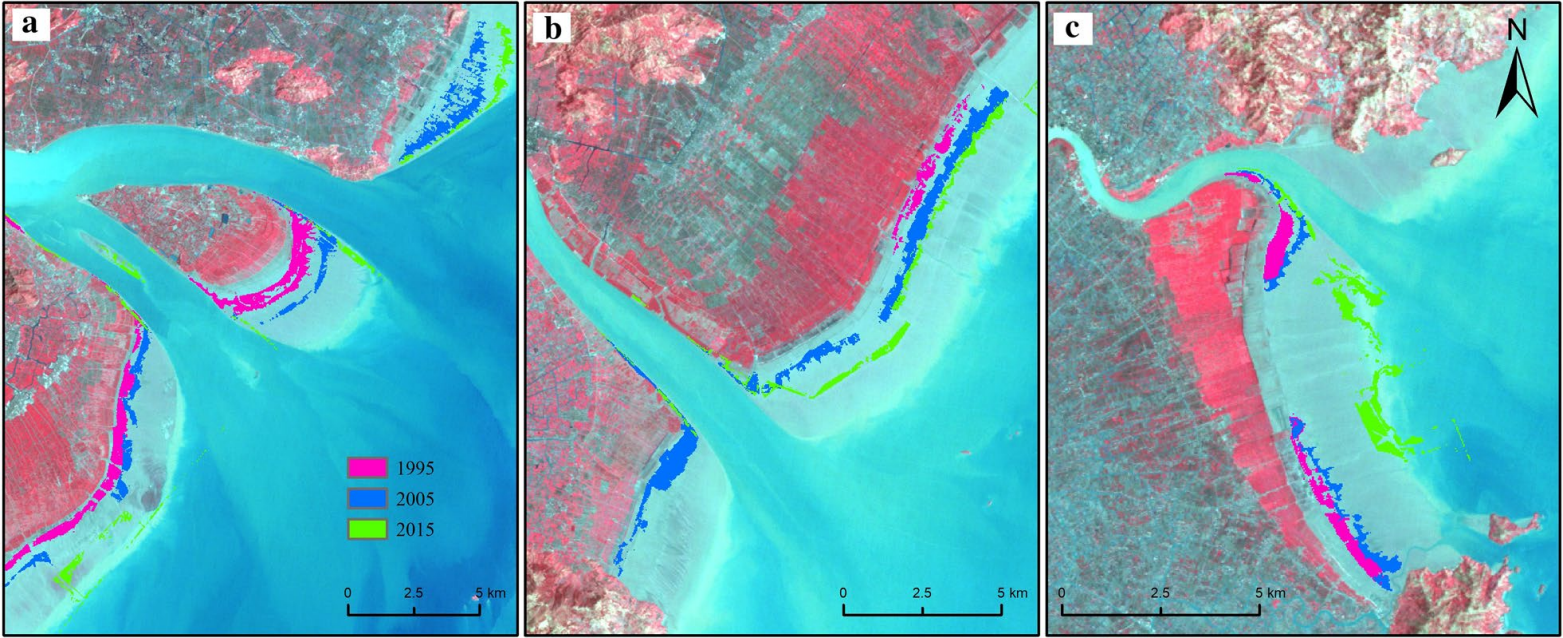
The expansion of *S. alterniflora* in the Ou River Estuary (**a**), Feiyun River Estuary (**b**) and Ao River Estuary (**c**). (Red, blue and green represent the distribution of *S. alterniflora* in 1995, 2005 and 2015, respectively)

Ou River Estuary, Feiyun River Estuary and Ao River Estuary are typical estuaries in the study area (Fig. 6). The sediment carried by the river accumulated on both sides of the estuaries; this accumulation, accompanied by high-intensity artificial reclamation, led to coastline expansion each year. *S. alterniflora* was distributed along the coast of the estuary area and moved outwards each year.

## Discussion

### Expansion dynamics of *S. alterniflora* in Zhejiang

*Spartina alterniflora* was planted in Taizhou in 1983, introduced to Wenzhou in 1989, and expanded to the Zhejiang coast in 1991. In the following decades, *S. alterniflora* expanded rapidly along the coast of Zhejiang. *S. alterniflora* has the largest area and fastest expansion rate in the bay and estuary areas. The sediments produced by the slow currents in these areas favor the growth and expansion of *S. alterniflora*. In contrast, the islands in Zhoushan and other places have large tidal currents and few tidal flats [36], making them unsuitable for the settlement of *S. alterniflora* and thus able to support only a small area of the species. Zhejiang coastal cities are densely populated, and dikes were built along the coast to protect cities and farmland. Therefore, in contrast to the expansion of terrestrial plants, which expand from inland areas toward the coast, the expansion of *S. alterniflora* is limited by dikes.

Although undergoing expansion in most regions, *S. alterniflora* has decreased in area in some regions over the past 30 years due to human control efforts, such as in Hangzhou Bay. On the one hand, sediment deposition led to the rapid accumulation of tidal flats outside the dikes, and *S. alterniflora* expanded accordingly. On the other hand, local residents eliminated *S. alterniflora* on the tidal flat and transformed the tidal flat into aquaculture ponds.

### Uncertainty in monitoring *S. alterniflora* invasion based on remote sensing

Due to the limited accessibility of tidal flats, it is difficult to pass through *S. alterniflora* areas. Field investigations are time consuming and laborious, making it difficult to conduct regional-scale surveys. Remote sensing technology has been widely used in monitoring studies of vegetation dynamics due to its repetitive acquisition of regional-scale images [27, 29]. However, there is still some uncertainty in monitoring the invasion of *S. alterniflora* based on remote sensing.

Landsat data are the most commonly used data for monitoring vegetation on a regional scale. However, the resolution of the data is relatively coarse (30 * 30 m), there are many mixed pixels, and vegetation areas less than 1000 m^2^ are difficult to distinguish. The edge of *S. alterniflora* communities is mixed with an *S. mariqueter* community or mangrove forest, and it is difficult to accurately extract *S. alterniflora* from remote sensing images. Studies have also used high-resolution images and unmanned aerial vehicle (UAV) images to extract areas of *S. alterniflora* with high precision. However, the cost of purchasing high-resolution images is high, and much work is needed to extract *S. alterniflora* areas on a regional scale.

Different classification methods also lead to certain uncertainties. It is widely believed that object-oriented methods are more accurate than pixel-based methods, but it is difficult to determine the optimal scale for object-oriented segmentation. Variables such as spectrum, texture, shape, and expert knowledge all contribute to the extraction of *S. alterniflora* and need to be used according to the data and actual conditions.

**Fig. 7:**
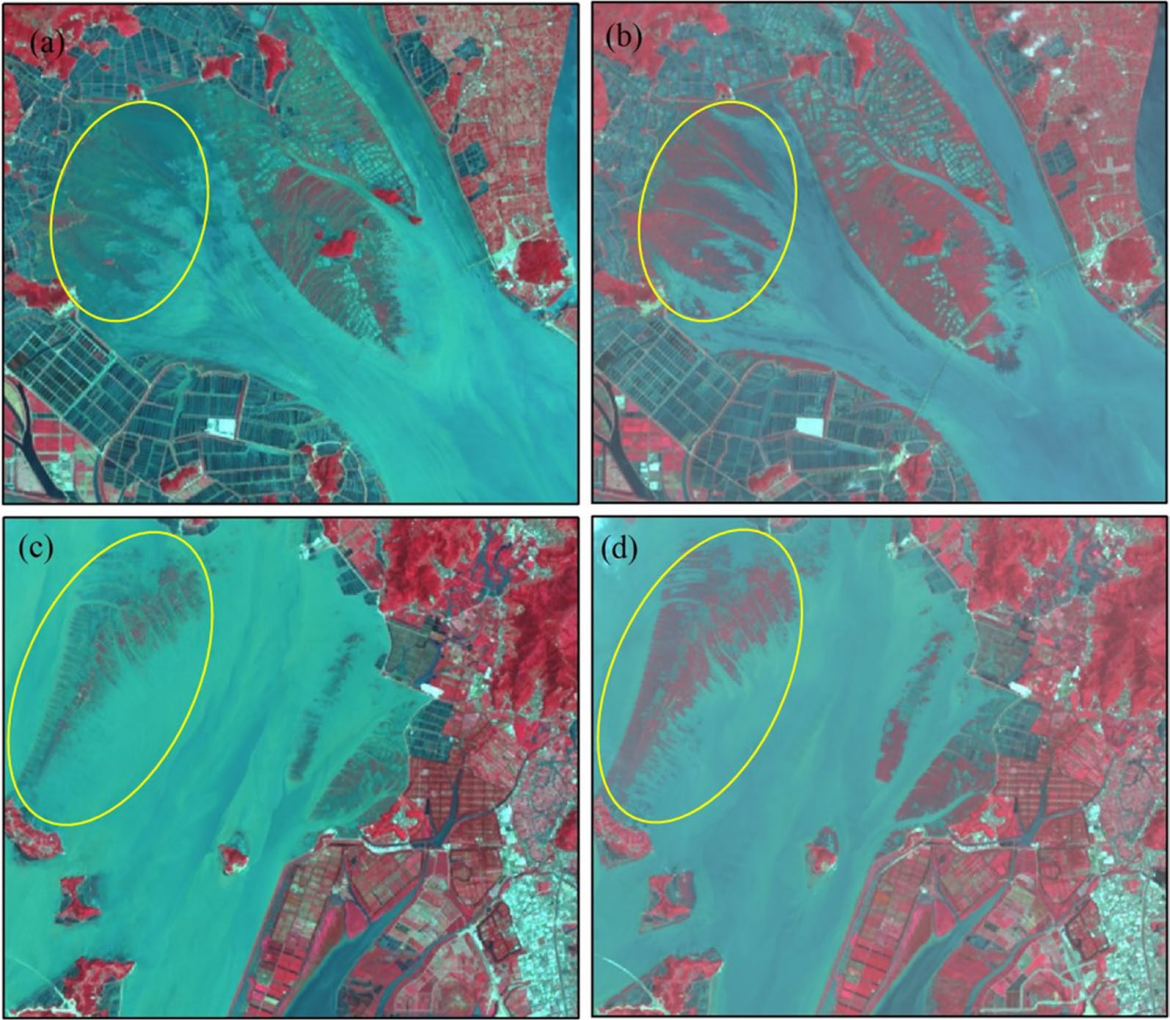
Remote sensing images of Sanmen Bay (**a**) and Yueqing Bay (**b**) under different tide conditions

In addition, tides are an important factor in the uncertainty of mapping *S. alterniflora*. *S. alterniflora* was concentrated in intertidal zones that are periodically submerged by seawater, and its area was affected by tidal water. In Fig. 7a, c are images of high-tide periods, when *S. alterniflora* was almost submerged in tidal water, and (b) and (d) are images of low-tide periods, when *S. alterniflora* grew well and was widely distributed. Remote sensing satellites have a defined visitation cycle and cannot obtain images of the same location every day. For example, the Landsat satellite takes images of the same place every 16 days. At the same time, if the weather conditions are not good, the images covered by clouds are almost impossible to use. When the study area is large (covering multiple scenes) and multiperiod changes are of interest, it is difficult to ensure that the acquired images are from the same low-tide period.

*Spartina alterniflora* rapidly expanded in tidal flats and was removed in some areas by local residents [12]. Therefore, results for *S. alterniflora* in the same year vary among studies, as do *S. alterniflora* mapping results in the same year. Lu et al. [1], Liu et al. [32] and Mao et al. [33] monitored *S. alterniflora* in China, but their results differed because of the study differences in the types of data acquired and in the study periods. It is difficult to say whose research results are more accurate because *S. alterniflora* has been changing dynamically and lacks reliable historical monitoring data. Future research may require more high-resolution data or the use of multiple data sources, as well as new methods such as machine learning, to more accurately monitor the invasion of *S. alterniflora.*

### The factors determining the invasion of *S. alterniflora*

Many factors affect the invasion of *S. alterniflora*. According to the literature, we summarize the main factors affecting the expansion of *S. alterniflora*. First, *S. alterniflora* has high tolerance to salt, flooding and temperature. *S. alterniflora* is a typical halophyte, with a salinity suitable for growth of 1% to 2%; this species can tolerate high salinity of up to 6%. In a saline environment, *S. alterniflora* adopts a salt-repelling strategy; it can also secrete salt [34]. The leaves of *S. alterniflora* are densely covered with stomata, and the highly developed aeration tissue transports oxygen to the underground part to relieve the oxygen deficiency caused by flooding [35]. This species can tolerate 12 h of flooding every day. When low- or high-temperature stress occurs, *S. alterniflora* can accumulate a large amount of soluble sugars.

Second, this species has a high reproductive capacity. The high seed yield and high germination rate of *S. alterniflora* facilitate its rapid expansion [36]. In addition to undergoing propagation by seed, *S. alterniflora* can grow axillary buds on the nodes of its stem and on underground stems, which can emerge from the soil surface and form new plants under suitable conditions [37]. Clonal reproduction facilitates the maintenance and renewal of populations as well as rapid proliferation and outbreaks [36, 38].

Third, the suitable habitat and lack of natural control mechanisms in this region promote *S. alterniflora*. There are no special growth requirements in terms of soil, and *S. alterniflora* can grow in clayey, loamy and silty soils. In China, silt and muddy tidal flats in coastal areas are rich in nutrients and are widespread. The tidal power, geology, climate, soil conditions and seawater salinity in these areas are quite suitable for *S. alterniflora* growth and reproduction. At present, there are no local, natural enemies that can control the growth and spread of *S. alterniflora* in the tidal flats of China. The imbalance of natural ecological competition has led to the uncontrolled proliferation of *S. alterniflora*. Due to the lack of natural control mechanisms, *S. alterniflora* can rapidly expand due to its strong growth ability [39].

Finally, intentional introduction and natural media promote *S. alterniflora* in this region. *S. alterniflora* has played an important role in reducing coastal erosion and promoting reclamation, and for this reason, its introduction was extended from Zhejiang, Jiangsu and Fujian Provinces to all coastal areas. Natural media such as tides and winds also influence the dispersal of *S. alterniflora* [40]. In addition, *S. alterniflora* can be unintentionally transferred through a variety of human activities, such as shipping and certain forms of land transport [39].

Muddy tidal flats are widely distributed along the coast of Zhejiang Province, providing suitable growth environments for *S. alterniflor*a. Summer currents flow from south to north, carrying the plants and seeds of *S. alterniflora* northward. Once *S. alterniflora* reaches new tidal flats, it can quickly settle and occupy the flats, grow extensively, and invade the native vegetation community.

## Conclusion

The rapid invasion of China’s coastal areas by *S. alterniflora* has serious consequences for local ecosystems. Accurate monitoring of the invasion is essential for coastal ecological protection. In this study, we proposed a simple and effective method to map the spatial distribution of *S. alterniflora* in Zhejiang Province in China, using a time series of Landsat images from 1985 to 2015. The results showed that the total area of *S. alterniflora* in Zhejiang was approximately 10,038.15 hm^2^ in 2015 and that the species was mainly distributed in bays, such as Hangzhou Bay, Yueqing Bay and Sanmen Bay, and estuaries, such as the Oujiang, Aojing, and Feiyun River estuaries. In recent decades, *S. alterniflora* has expanded rapidly, with the highest expansion rate between 1995 and 2005, reaching 463.64 hm^2^/year. During the invasion from south to north, the extent of expansion varied among regions, with Ningbo exhibiting the greatest expansion. This study provides multitemporal distribution data for the study of *S. alterniflora* invasion in Zhejiang, revealing the temporal and spatial dynamics of the invasion process and helping the government control *S. alterniflora*.

## Data Availability

The datasets generated and analyzed during the current study are available in the Dryad repository at https://datadryad.org/stash/share/b_OmhaXulXQdNvaCnAiWHSwuoyQNi64ws5Ol_tbPUfE.

## Abbreviations

*S. alterniflora*: *Spartina alterniflora*
TM: Landsat Thematic Mapper
OLI: Operational Land Imager
UTM: Universal Transverse Mercator
NDVI: Normalized difference vegetation index
UAV: Unmanned aerial vehicle
NASA: National Aeronautics and Space Administration
